# Electrochemical Determination of Doxorubicin in the Presence of Dacarbazine Using MWCNTs/ZnO Nanocomposite Modified Disposable Screen-Printed Electrode

**DOI:** 10.3390/bios15010060

**Published:** 2025-01-17

**Authors:** Somayeh Tajik, Hadi Beitollahi, Fariba Garkani Nejad, Zahra Dourandish

**Affiliations:** 1 Research Center of Tropical and Infectious Diseases, Kerman University of Medical Sciences, Kerman 76169-13555, Iran; 2 Environment Department, Institute of Science and High Technology and Environmental Sciences, Graduate University of Advanced Technology, Kerman 76318-85356, Iran; h.beitollahi@kgut.ac.ir (H.B.); garkani@sci.uk.ac.ir (F.G.N.); dourandish@sci.ac.ir (Z.D.)

**Keywords:** MWCNTs/ZnO nanocomposite, doxorubicin, dacarbazine, electrochemical sensor, screen-printed carbon electrode, voltammetric determination

## Abstract

In the current work, the MWCNTs/ZnO nanocomposite was successfully synthesized using simple method. Then, FE-SEM, XRD, and EDX techniques were applied for morphological and structural characterization. Afterward, a sensitive voltammetric sensor based on modification of a screen-printed carbon electrode (SPCE) using MWCNTs/ZnO nanocomposite was developed for the determination of doxorubicin in the presence of dacarbazine. To evaluate the electrochemical response of the MWCNTs/ZnO/SPCE towards doxorubicin, cyclic voltammetry (CV) was applied. The MWCNTs/ZnO nanocomposite showed a significant synergistic effect on the electrochemical response of the electrode for the redox reaction of doxorubicin. Also, the MWCNTs/ZnO/SPCE demonstrated an enhanced sensing platform for the quantification of doxorubicin, obtaining a detection limit (LOD) of 0.002 µM and a sensitivity of 0.0897 µA/µM, as determined by differential pulse voltammetry (DPV) within a linear range from 0.007 to 150.0 µM. Also, the MWCNTs/ZnO nanocomposite-modified SPCE showed high electrochemical activities towards the oxidation of doxorubicin and dacarbazine with peak-potential separation of 345 mV, which is sufficient for doxorubicin determination in the presence of dacarbazine. Also, the MWCNTs/ZnO nanocomposite-modified SPCE presented reproducible and stable responses to determine doxorubicin. Finally, the developed platform demonstrated a successful performance for doxorubicin and dacarbazine determination in real samples, with recovery in the range of 97.1% to 104.0% and relative standard deviation (RSD) from 1.8% to 3.5%.

## 1. Introduction

Cancer is one of the common diseases that is considered one of the main concerns and causes of death worldwide. Cancer is a disease in which the cells of body grow and divide uncontrollably and may spread to other tissues. In recent years, chemotherapy has been one of the most widely used treatment methods for cancer, offering effective results. Chemotherapy is a treatment method for cancer that uses chemical drugs to destroy cancer cells or control their growth [1]. Like any cancer treatment, chemotherapy has many side effects. Chemotherapy can damage and destroy normal cells, which is considered one of the main problems of chemotherapy. Therefore, due to the side effects of chemotherapy drugs, it is very necessary to monitor the concentration of these drugs to improve their effectiveness and reduce side effects. Doxorubicin, known by the trade name of Adriamycin, is an effective antitumor agent belonging to the anthracyclines^,^ family of antibiotics [2,3]. It has been extensively utilized in the treatment of a wide range of cancers, including breast cancer, lung cancer, acute lymphoblastic leukemia, soft tissue sarcomas, Hodgkin’s lymphoma, and so forth [4,5]. There are two proposed mechanisms through which doxorubicin exerts its action on cancer cells [6]: (I) intercalation into the double-stranded DNA helix and disruption of topoisomerase-II-mediated DNA repair, and (II) generation of free radicals that cause damage to cellular membranes, DNA, and proteins. However, numerous side effects have been reported in the human body during doxorubicin therapy, including cardiotoxicity, nausea or vomiting, pain, and emergence of drug resistance [7]. Hence, the development of a reliable and fast method for analyzing doxorubicin levels to guide dosage during the chemotherapy process is very important. Dacarbazine [5-(3,3-Dimethyl-1-triazenyl)imidazole-4-carboxamide; DTIC], a crucial antitumor agent, has been demonstrated to effectively treat various types of cancers, such as soft tissue sarcoma, lung carcinoma, Hodgkin’s lymphoma, malignant melanoma, and childhood solid tumors [8,9]. Dacarbazine functions as an alkylating agent after metabolization in the liver and produces highly reactive intermediates with alkylating activity. It causes methylation of the DNA bases, which inhibits DNA, RNA, and protein synthesis [10,11]. Despite the effectiveness of this drug in the treatment of cancers, side effects include nausea, vomiting, constipation, low white blood cell counts, and other effects during chemotherapy treatment [12]. The recent progress in chemotherapy is the utilization of combination drug therapy, where multiple chemotherapeutic agents are administered simultaneously. This approach aims to enhance efficacy, create synergistic effects, and minimize side effects. The simultaneous use of doxorubicin and dacarbazine in combination therapy shows promise as an effective treatment regimen for metastatic sarcomas [13]. Therefore, the simultaneous determination of doxorubicin and dacarbazine in pharmaceutical and biological compounds is crucial for quality control and diagnosis of diseases. In this regard, several techniques, such as liquid chromatography coupled with mass spectrometry (MS), high-performance liquid chromatography, chemiluminescence, capillary electrophoresis, and fluorimetry, have been developed and reported to detect doxorubicin and dacarbazine in pharmaceutical and biological samples [14,15,16,17,18,19,20]. Some of these reported techniques have shown good efficiency, high sensitivity and selectivity. However, these analytical methods are not suitable for performing fast, routine, and on-site analysis, owing to the requirements of expensive and sophisticated instruments, time-consuming processing and sampling, skilled and expert operators, etc. Therefore, there is a growing interest in detection methods that are fast, simple, sensitive, selective, and accurate compared to other methods. 

Electrochemical sensing methods are very efficient and suitable, as they provide simple and sensitive measurements with no requirement for complicated sample pre-preparations, and they also offer relatively good selectivity [21,22,23,24,25,26,27,28,29,30,31]. However, one of the basic challenges and limitations of using conventional electrodes that needs to be solved is their poor sensitivity and selectivity towards the detection of electroactive compounds. As a result, the modification of the surface of electrodes is highly desirable to design novel and very efficient electrochemical sensors [32,33,34,35,36,37,38,39]. In the fabrication of electrochemical sensors, research has focused on the use of materials with high surface area, high porosity, high conductivity, low-cost, high stability, etc. [40,41,42,43,44,45].

Nanotechnology is one of the novel and rapidly developing technologies within a wide range of fields [46,47,48,49,50,51,52,53,54,55]. Also, nanoscience as one of the basic sciences provides very high capabilities can be highly effective in the progress and improvement of other sciences. In particular, nanostructured materials are considered to be efficient materials that make possible the fabrication of very sensitive and efficient electrochemical sensors by increasing the surface area of electrodes and enhancing the electron transfer rate [56,57,58,59,60,61,62]. The distinctive characteristics of metal oxide nanomaterials, including biocompatibility, optical properties, and catalytic activities have garnered significant attention for their use in sensing applications [63,64]. Zinc oxide nanoparticles (ZnO NPs) are a type of transition metal oxide with unique properties such as non-toxicity, semiconductor behavior, thermal stability, and electrochemical activity, making them suitable for various applications. ZnO NPs offer various advantages for sensing platforms, including efficient surface modification, size distribution, fast electron transfer kinetics, electrochemical activity, large surface-to-volume ratio, and biocompatibility [65,66]. ZnO NPs are considered superior functional materials due to the numerous active sites present on their surface, which can efficiently bond with various carbon-supporting materials such as graphene oxide (GO), graphite, carbon ribbons, carbon nanotubes (CNTs), carbon microspheres, and carbon black [67,68]. The exceptional structural, electronic, and mechanical properties of CNTs have been well-established, rendering them highly appealing for a diverse range of applications. Due to their nano-scale dimensions, electronic structure, and the presence of topological defects on their surfaces, CNTs possess excellent capacitive characteristics that facilitate electron transfer in reactions when utilized as electrode materials. [69,70,71]. Thanks to these outstanding properties of CNTs, they have been extensively and effectively employed in the development of electrochemical sensors. The combination of the complementary properties of CNTs and ZnO NPs results in numerous exceptional electrical, mechanical, and electrochemical properties that cannot be individually achieved by either CNTs or ZnO NPs [72,73].

Therefore, the objective of the current study was to investigate the development of a MWCNTs/ZnO nanocomposite modified SPCE for the simple, rapid, and sensitive determination of doxorubicin in the presence of dacarbazine. The MWCNTs/ZnO/SPCE sensor exhibited good performance for the redox reaction of doxorubicin, which presented a broad linear range, a low detection limit, and strong electrochemical properties. Also, the performance of the developed MWCNTs/ZnO/SPCE was evaluated by monitoring doxorubicin concentrations in the presence of dacarbazine by DPV. Additionally, the applicability of this sensor for real sample analysis was successfully investigated by analyzing real samples using the standard addition method. The novelty of the present study relates to the use of MWCNTs/ZnO nanocomposite modified SPCE for the electrochemical determination of doxorubicin in the presence of dacarbazine.

## 2. Experimental

### 2.1. Materials and Reagents

All materials were of analytical reagent grade (with higher purity) and were employed in the present work without further purifications.

### 2.2. Instrumentation

The morphological analysis and structural characterization of the MWCNTs/ZnO nanocomposite, including field-emission scanning electron microscopy (FE-SEM), energy-dispersive X-ray spectroscopy (EDX), and X-ray diffraction, (XRD) were performed by using a field-emission scanning electron microscope (MIRA3, TESCAN, Brno, Czech Republic) equipped with an EDX detector and X-ray powder diffractometer (X′Pert Pro, Panalytical, Almelo, The Netherlands).

The chronoamperometry, CV, and DPV measurements were carried out using a potentiostat-galvanostat Autolab (PGSTAT 302N, Metrohm, Herisau, Switzerland). In this study, a DRP-110 model of SPCE was purchased from Dropsens (DropSens, Oviedo, Spain). The Metrohm-713 pH meter (Herisau, Switzerland) was employed for the pH measurements of phosphate buffers. 

### 2.3. Synthesis of MWCNTs/ZnO Nanocomposite

The MWCNTs/ZnO nanocomposite was synthesized with slight modifications to the procedure described in the previous study by Ranjithkumar et al. [74]. The MWCNT–COOH (65 mg) was firstly dispersed in deionized water (50 mL) in a round-bottomed flask by sonication (40 min) to obtain a homogenous suspension. Then, 0.148 g of Zn(NO_3_)_2_.6H_2_O was added into the MWCNT suspension, and the mixture was stirred at 120 °C (3 h). After three hours, a specific amount of NaOH (0.2 g) was dissolved in 12.5 mL of deionized water and added dropwise to the above solution. Following this step, 0.07 g of hexamine was added, and the reflux process continued for 3 h at 120 °C. Finally, the synthesized nanocomposite was collected, washed multiple times with deionized water and ethanol, and dried under vacuum conditions at 80 °C for 10 h.

### 2.4. Preparation of the Modified SPCE

To modify the SPCE surface, the MWCNTs/ZnO nanocomposite (1.0 mg) was initially suspended in deionized water (1.0 mL) and subjected to ultrasonic treatment for at least 20 min. The prepared suspension of nanocomposite (3.0 µL) was then drop-casted and air-dried on the working electrode of the SPCE.

## 3. Results and Discussion

### 3.1. Characterization of the MWCNTs/ZnO Nanocomposite

**Figure 1:**
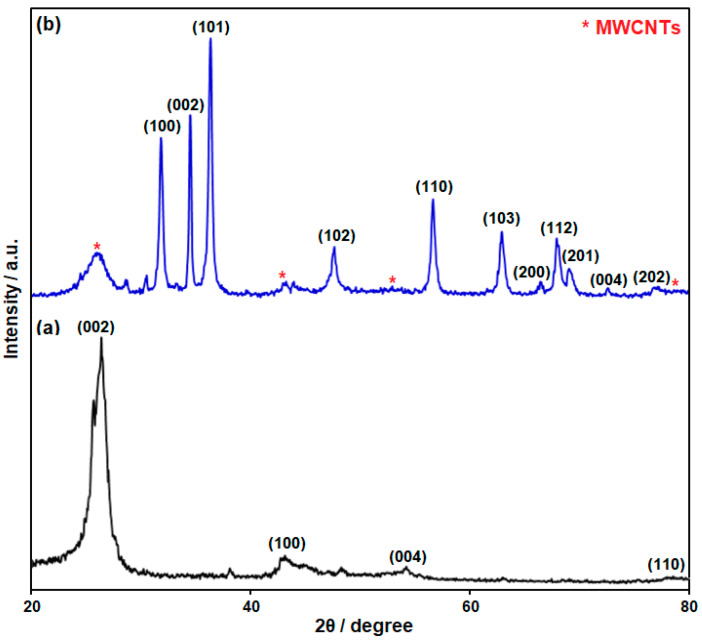
XRD patterns of MWCNTs-COOH (pattern (**a**)) and MWCNTs/ZnO nanocomposite (pattern (**b**)).

The XRD patterns of the MWCNTs-COOH and MWCNTs/ZnO nanocomposite are displayed in Figure 1, which provides details about the crystalline structure. The MWCNTs-COOH showed diffraction peaks at 26.2, 42.8, 53.9, and 78.1°, which were attributed to diffraction from the (002), (100), (004), and (110) planes of MWCNTs-COOH, respectively (Figure 1a). The observation of these diffraction peaks demonstrates the hexagonal graphite structure of MWCNTs. In the XRD pattern of the prepared nanocomposite (Figure 1b), the diffraction peaks located at 31.7, 34.4, 36.3, 47.5, 56.6, 62.8, 66.4, 67.9, 69.1, 72.5, and 77.0° assigned to diffractions from (100), (002), (101), (102), (110), (103), (200), (112), (201), (004), and (202) planes of ZnO, respectively. All the diffraction peaks indexed as the hexagonal wurtzite structure of ZnO. Furthermore, the XRD pattern of the MWCNTs/ZnO nanocomposite also exhibited the characteristic peaks of MWCNTs-COOH. The presence of these characteristic peaks confirmed the formation of the MWCNTs/ZnO nanocomposite.

**Figure 2:**
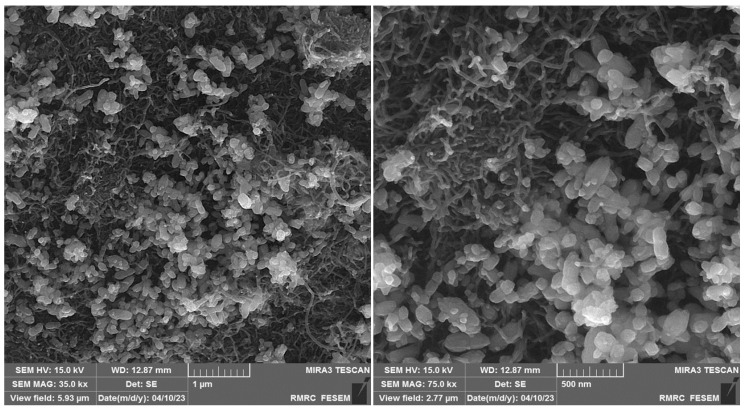
FE-SEM images of MWCNTs/ZnO nanocomposite.

Figure 2 displays the morphological characteristics of the MWCNTs/ZnO nanocomposite, which were evaluated using FE-SEM images. As can be seen from Figure 2, a network of MWCNTs is interwoven among the ZnO NPs, indicating the successful formation of the MWCNTs/ZnO nanocomposite.

**Figure 3:**
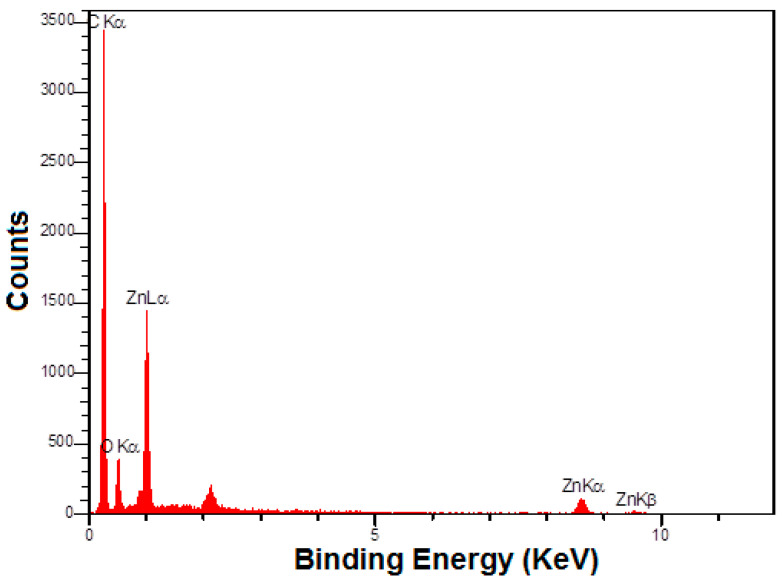
The EDS spectrum of MWCNTs/ZnO nanocomposite.

Furthermore, the elemental analysis (Figure 3 (EDX spectrum)) revealed the presence of C, O, and Zn elements.

### 3.2. Comparison of the Electrochemical Behavior of Doxorubicin on the Surface of Unmodified and Modified SPCEs

**Figure 4:**
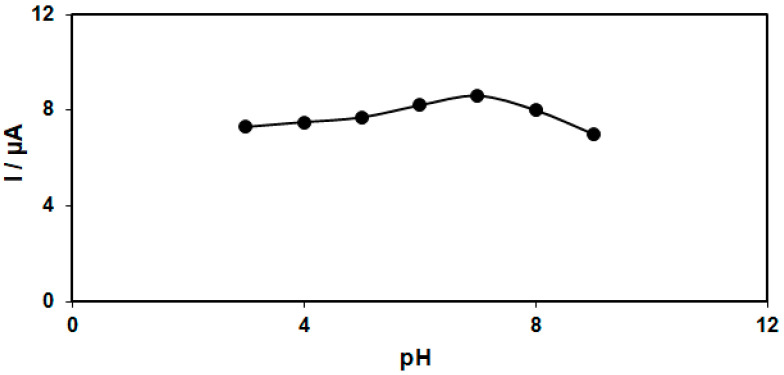
Plot of the oxidation peak currents of doxorubicin (90.0 µM) obtained from DPV of the modified electrode as a function of pH solution (3.0, 4.0, 5.0, 6.0, 7.0, 8.0 and 9.0).

**Scheme 1:**
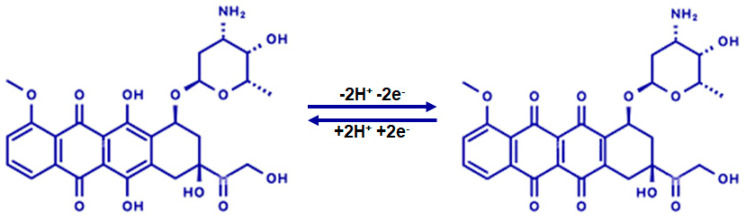
The probable redox mechanism for doxorubicin.

The most influential parameter in the electroanalysis of compounds is pH. In order to investigate this effect, DPV studies were taken in phosphate buffer (0.1 M-PBS) (at different pH values ranging from 3.0 to 9.0) containing 90.0 µM doxorubicin (Figure 4). According to the findings, the increase in pH value (from 3.0 to 7.0) induced a significant rise in the anodic peak current (Ipa) value, followed by a decrease at pH values higher than pH 7.0 (pH 8.0 and pH 9.0). The highest electrochemical response of doxorubicin was observed in PBS 0.1 M at pH 7.0. Therefore, the electrochemical studies and evaluation of the analytical application of the designed sensor were carried out in buffer solutions at pH 7.0. The proposed mechanism for the electrochemical (redox) reaction of doxorubicin is displayed in Scheme 1.

**Figure 5:**
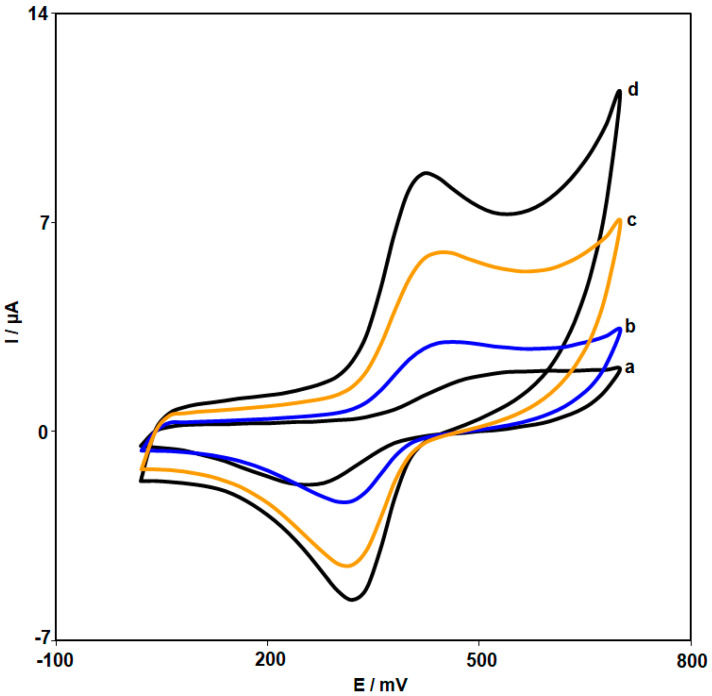
CV responses recorded for unmodified SPCE (**a**), ZnO NPs/SPCE (**b**), MWCNTs/SPCE (**c**), and MWCNTs/ZnO/SPCE (**d**) in 0.1 M phosphate buffer (pH 7.0) containing 90.0 µM doxorubicin at a scan rate of 50 mV/s.

CV tests were also carried out, to evaluate the performance of the MWCNTs/ZnO nanocomposite in the electrochemical determination of doxorubicin. In this test, the CV responses of an unmodified SPCE (curve a), the ZnO NPs/SPCE (curve b), the MWCNTs/SPCE (curve c), and the MWCNTs/ZnO/SPCE (curve d) were recorded in 0.1 M PBS at pH 7.0 containing 90.0 µM of doxorubicin. The corresponding voltammograms are presented in Figure 5. As can be seen, unmodified and modified electrodes showed well-defined redox peaks (anodic/cathodic peaks) for doxorubicin. However, on the SPCE modified with MWCNTs/ZnO nanocomposite, a considerable effect in detecting doxorubicin was observed. The unmodified SPCE demonstrated a weak voltammetric response for the redox reaction of doxorubicin, leading to relatively lower values of currents. Through modification of the SPCE surface with ZnO NPs, the redox peak currents of doxorubicin were enhanced, which demonstrated the effective performance of ZnO NPs. Also, the redox peak currents of doxorubicin on the MWCNTs-modified SPCE were enhanced compared to the redox peak currents on the ZnO NPs/SPCE. Conversely, the MWCNTs/ZnO nanocomposite modified SPCE displayed an enhanced voltammetric response towards doxorubicin compared to other electrodes. In addition, the anodic peak of doxorubicin was observed at lower potentials in the MWCNTs/ZnO/SPCE compared to unmodified SPCE and other modified electrodes. The comparison of these CVs indicated that the MWCNTs/ZnO/SPCE is more sensitive towards doxorubicin compared to other SPCEs, which can be related to the favorable effects of MWCNTs and ZnO NPs, as well as their synergistic effects.

### 3.3. Effect of Scan Rate

**Figure 6:**
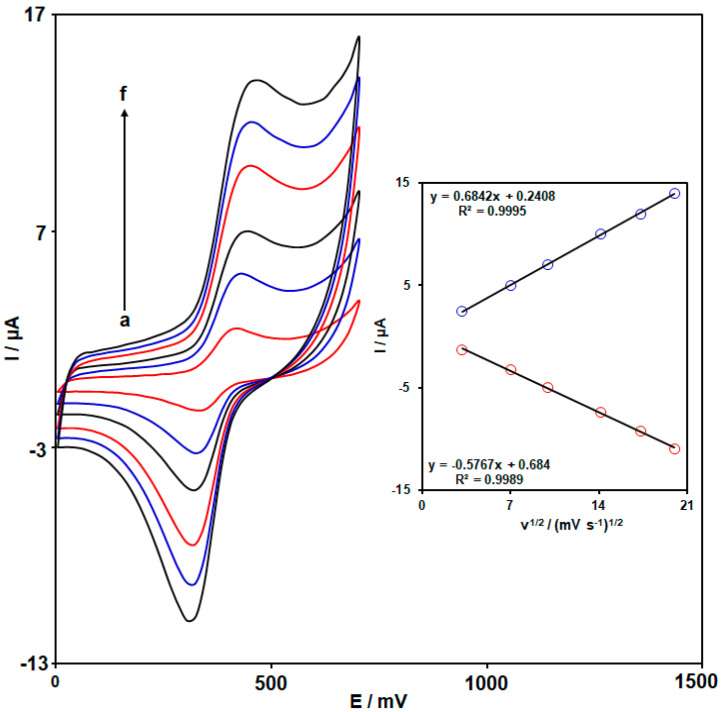
CV responses of the MWCNTs/ZnO/SPCE in detecting 50.0 µM doxorubicin in phosphate buffer 0.1 M at pH 7.0 at various scan rates (from a to f: 10 mV s^−1^, 50 mV s^−1^, 100 mV s^−1^, 200 mV s^−1^, 300 mV s^−1^, and 400 mV s^−1^). Inset: linear plots of the redox peak currents vs. υ^1/2^.

To evaluate the electrochemical redox mechanism of doxorubicin on the MWCNTs/ZnO/SPCE, the CV responses of 50.0 µM doxorubicin in 0.1 M PBS-pH 7.0 were recorded at various scan rates (10 mV/s to 400 mV/s) (Figure 6). The CVs are presented in Figure 6, demonstrating a distinct increase in the redox peak currents when increasing the scan rates. Additionally, the strong linear dependence between the Ipa vs. υ^1/2^ and Ipc vs. υ^1/2^ was obtained using the following equations: (Ipa = 0.6842 υ^1/2^ (mV s^−1^)^1/2^ + 0.2408 (R^2^ = 0.9995)) and (Ipc = −0.5767 υ^1/2^ (mV s^−1^)^1/2^ + 0.684 (R^2^ = 0.9989)), respectively (Figure 6 (Inset)). This demonstrated that the electron transfer in the redox reaction of doxorubicin was under the control of the diffusion phenomenon. 

### 3.4. Chronoamperometric Studies

**Figure 7:**
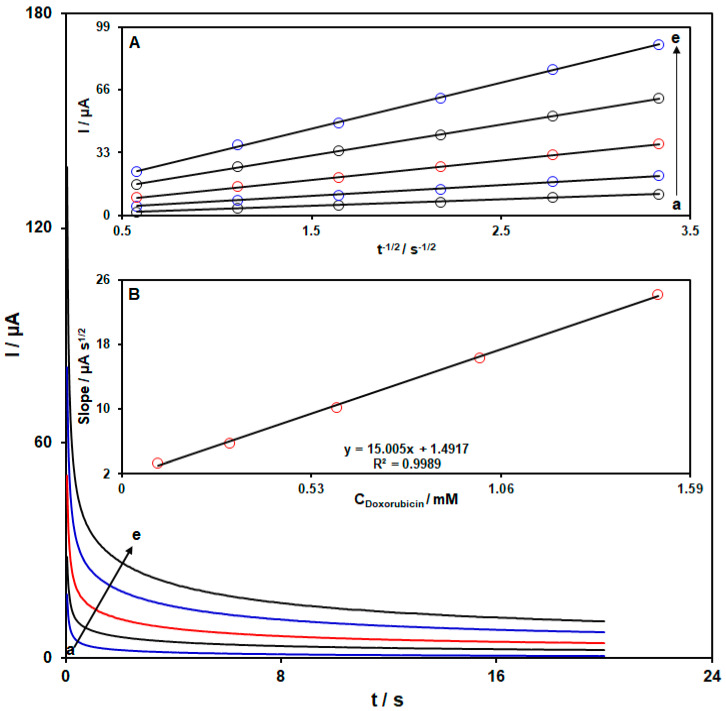
Chronoamperometric responses of MWCNTs/ZnO/SPCE for detecting various concentrations (from a to e: 0.1 mM, 0.3 mM, 0.6 mM, 1.0 mM, and 1.5 mM) of doxorubicin in 0.1 M phosphate buffer at pH 7.0. Inset (**A**) demonstrates the plot of current response vs. t^−1/2^ obtained from chronoamperograms. Inset (**B**) demonstrates the plot of slopes from the obtained straight lines vs. doxorubicin concentration.

Chronoamperometry technique is often applied to measure the diffusion coefficient of electroactive species. In this technique, a constant potential is applied to the working electrode, and the dependence of current on time is tracked. The chronoamperograms displayed in Figure 7 were recorded at a constant potential of 470 mV for the MWCNTs/ZnO/SPCE in 0.1 M of phosphate buffer containing various concentrations (0.1, 0.3, 0.6, 1.0, and 1.5 mM) of doxorubicin. The current response is described by the Cottrell equation:

I = nFACD^1/2^π^−1/2^t^−1/2^

where:

I = current response (µA)
n = the number of electrons transferred
F = Faraday’s constant (96,485 C/mol)
C = bulk concentration of doxorubicin (mol/cm^3^)
D = diffusion coefficient (cm^2^/s)
t = time (s).

Figure 7 (Inset A) depicts a linear dependence between the current response and t^−1/2^ for each concentration of doxorubicin, showing that the electrochemical reaction of target analyte is often controlled by the diffusion process. The resulting slopes were plotted against the concentrations of doxorubicin (See Inset B of Figure 7). This plot exhibits a relatively good linear dependence within the tested concentration range of doxorubicin (correlation coefficient (R^2^) = 0.9989). From the resulting slope of this plot and Cottrell equation, the D value was estimated as 1.9 × 10^−5^ cm^2^/s for doxorubicin.

### 3.5. Quantitative Analysis of Doxorubicin

**Figure 8:**
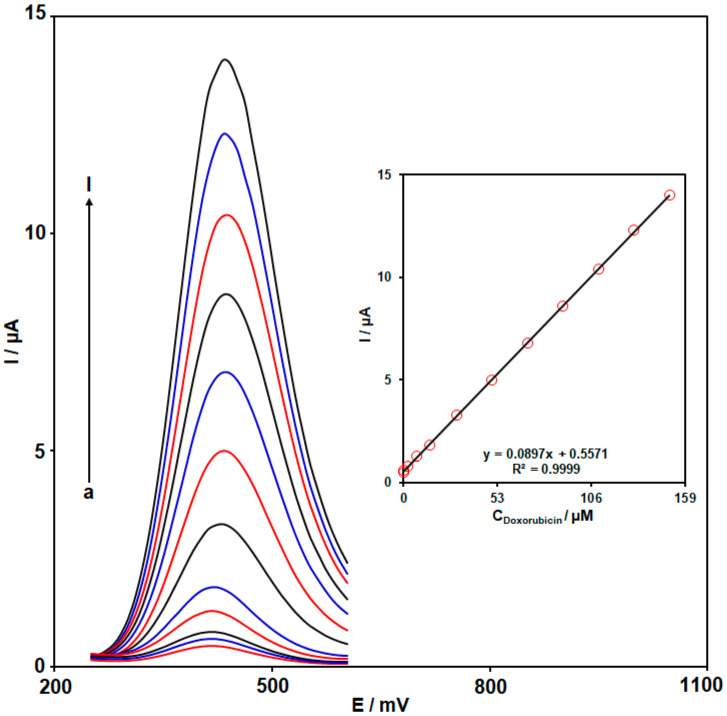
DPV responses of MWCNTs/ZnO/SPCE when measuring different concentrations (from a to l: 0.007 µM, 0.1 µM, 2.5 µM, 7.5 µM, 15.0 µM, 30.0 µM, 50.0 µM, 70.0 µM, 90.0 µM, 110.0 µM, 130.0 µM, and 150.0 µM) of doxorubicin in phosphate buffer at pH 7.0. Inset: Corresponding calibration curve.

Figure 8 exhibits the voltammograms from the analysis conducted to assess the performance of the MWCNTs/ZnO nanocomposite modified SPCE in detecting various concentrations of doxorubicin. From the obtained DPVs, a clear response towards doxorubicin was observed, and the current response values of doxorubicin increased by increasing its concentrations, which ranged from 0.007 µM to 150.0 µM. Then, the values of Ipa obtained from DPVs were plotted against the various concentrations of doxorubicin. The corresponding calibration curve is given in Figure 8 (Inset). The calibration plot presents strong linearity within the broad range of measurements from 0.007 to 150.0 µM (linear regression equation of Ipa = 0.0897C_doxorubicin_ + 0.5571 with R^2^ = 0.9999). In addition, the LOD was calculated to be 0.002 µM for doxorubicin. The sensing performance of the developed MWCNTs/ZnO nanocomposite modified SPCE-based electrochemical sensor in this work and some previously reported sensors for doxorubicin determination are presented in Table 1. As demonstrated in Table 1, the performance of the MWCNTs/ZnO/SPCE sensor to determine doxorubicin is better than or comparable with other sensors.

### 3.6. DPV Measurements of Doxorubicin in the Presence of Dacarbazine at MWCNTs/ZnO/SPCE

**Figure 9:**
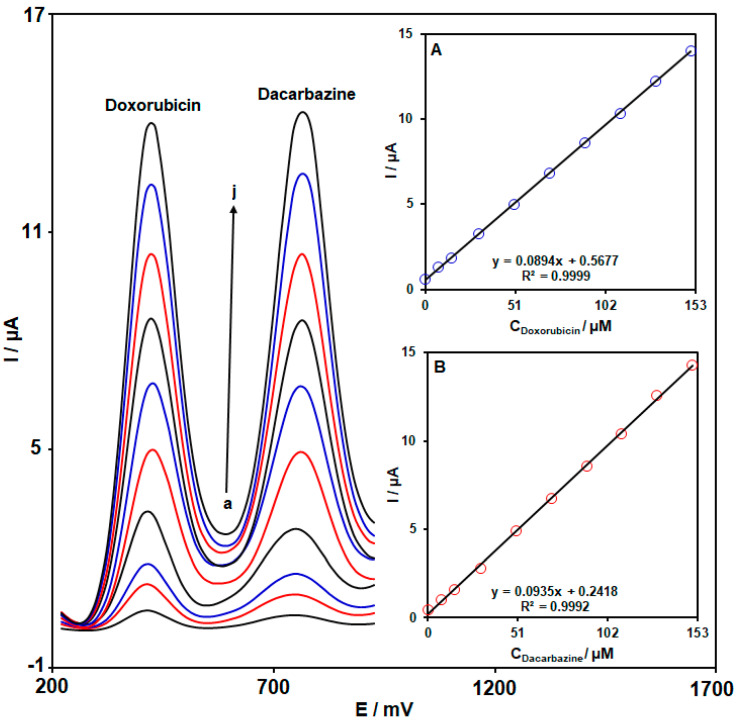
DPV responses of MWCNTs/ZnO/SPCE sensor in 0.1M PBS (pH 7.0) containing various concentrations of doxorubicin and dacarbazine (voltammogram a to voltammogram j corresponded to 0.1 µM + 0.1 µM, 7.5 µM + 7.5 µM, 15.0 µM + 15.0 µM, 30.0 µM + 30.0 µM, 50.0 µM + 50.0 µM, 70.0 µM + 70.0 µM, 90.0 µM + 90.0 µM, 110.0 µM + 110.0 µM, 130.0 µM + 130.0 µM, and 150.0 µM + 150.0 µM doxorubicin and dacarbazine, respectively). Insets: Corresponding calibration plots for doxorubicin (Inset (**A**)) and dacarbazine (Inset (**B**)).

The electrochemical determination of doxorubicin at the MWCNTs/ZnO/SPCE sensor was investigated in 0.1 M PBS (pH 7.0) in the presence of dacarbazine by DPV (Figure 9). Based on the obtained voltammograms, two well-resolved oxidation peaks were detected at 420 mV and 765 mV for doxorubicin and dacarbazine, respectively. A significant increase in the values of peak current was observed, with the increasing concentration of doxorubicin in a range from 0.1 µM to 150.0 µM and the increasing concentration of dacarbazine in a range from 0.1 µM to 150.0 µM, respectively. Then, the Ipa vs. concentrations of doxorubicin and dacarbazine were plotted, and the correlation equations for doxorubicin and dacarbazine were derived from the calibration plots as shown in Inset A and Inset B of Figure 9. Additionally, the corresponding slope of the calibration plot for doxorubicin (Inset A) confirms the high sensitivity of the prepared sensor towards doxorubicin.

### 3.7. Reproducibility, Repeatability, and Stability Studies

The effective performance of a sensor also has a significant dependence on its high reproducibility, repeatability, and stability in detecting the target compound. Therefore, in the developing of electrochemical sensors and biosensors, it is very important to consider reproducibility, repeatability, and stability as essential factors. The reproducibility of the MWCNTs/ZnO/SPCE sensor in the detection of 50.0 µM doxorubicin in PBS (0.1 M—pH = 7.0) was investigated using six electrodes prepared in the same way. The electrochemical responses of these electrodes were recorded by using DPV towards doxorubicin determination. The responses of these sensors were similar, matching with each other, and the relative standard deviation (RSD) was estimated to be 3.9%. It was shown that the modified SPCE had a good reproducibility. The repeatability of the modified SPCE developed was also assessed with 10 continuous measurements of a sample containing 50.0 µM doxorubicin in PBS (0.1 M—pH = 7.0) using the same sensor. No significant decrease in the current responses was observed in the measurements. The findings indicated that the current response of the modified SPCE was retained at 95.7% of its initial value. It was demonstrated that the developed sensor has repeatable responses. In addition, the stability of the MWCNTs/ZnO/SPCE was evaluated by storing it at ambient temperature over a period of 15 days. The response of this sensor was recorded every 5 days for 15 days through the measurement of 50.0 µM doxorubicin in PBS (0.1 M—pH = 7.0). After 15 days, the high stability of the sensor was confirmed, with a slight decrease in the DPV response of the modified SPCE of less than 4.2%.

### 3.8. Interference Studies

In the electroanalysis of compounds, the response of the electrochemical sensor is affected by the presence of other compounds, which are known as interfering species. Therefore, the ability of the MWCNTs/ZnO/SPCE sensor to determine doxorubicin in the presence of common ions was evaluated using the DPV technique. It was observed that there was no obvious interference from investigated ions including K^+^, Na^+^, Mg^2+^, Ca^2+^, NH_4_^+^, Cl^−^, CO_3_^2−^, and SO_4_^2−^ (the relative signal change of doxorubicin in the presence of even 200 times excess concentration of these species was less than ±5%). At the same time, the influence of epirubicin and daunorubicin on the determination of doxorubicin was also assessed. From the obtained results, it was found that the presence of these species affected the response of the MWCNTs/ZnO/SPCE sensor for the determination of doxorubicin.

### 3.9. Application of the MWCNTs/ZnO/SPCE for Analysis of Doxorubicin and Dacarbazine in Real Samples

To evaluate the possible ability of the developed sensor to make quantitative determinations of doxorubicin and dacarbazine in doxorubicin and dacarbazine injection samples, the MWCNTs/ZnO/SPCE was utilized to conduct DPV measurements after adding known concentrations of doxorubicin and dacarbazine to the samples. The results from the analysis of spiked samples are presented in Table 2. The obtained findings in Table 2 reveal the effective ability of the MWCNTs/ZnO/SPCE sensor to determine doxorubicin and dacarbazine in real samples, with recovery values ranging from 97.1% to 104.0%. Furthermore, the RSD ranged between 1.8% and 3.5%, illustrating the precision of the sensor in the analysis of doxorubicin and dacarbazine in real samples.

## 4. Conclusions

In summary, we have reported an electrochemical sensor for doxorubicin determination in the presence of dacarbazine using SPCE modified with MWCNTs/ZnO nanocomposite. The obtained findings reveal that the combination of MWCNTs and ZnO NPs significantly improve the response of the SPCE for doxorubicin determination. The linear response between the Ipa and doxorubicin concentrations was observed to be in the 0.007 to 150.0 µM range, along with a detection limit of 0.002 µM. Also, the modified SPCE demonstrated well-defined peak-to-peak separation for the determination of doxorubicin in the presence of dacarbazine without the two interfering with each other. Additionally, the repeatable response, long-term stability, and reproducibility of the MWCNTs/ZnO nanocomposite modified SPCE were observed for doxorubicin determination. In addition, the MWCNTs/ZnO/SPCE sensor enabled the determination of doxorubicin and dacarbazine in real samples. The satisfactory and accurate results were obtained using the application of doxorubicin and dacarbazine in injection sample analysis.

## Figures and Tables

**Table 1:** Comparison between different electrochemical sensors for doxorubicin determination and the sensor developed in this work.

Electrochemical Sensor	Electrochemical Method	Linear Range	LOD	Ref.
Bird nest-like nano-structured NiCo_2_O_4_/SPE	DPV	0.01 to 600.0 μM	9.4 nM	[2]
Vertically ordered mesoporous silica film/electrochemically pretreated glassy carbon electrode (GCE)	DPV	0.5 nM to 23 μM	0.2 nM	[3]
Pencil graphite electrode (PGE)	CV	10 to 60 µM	9.9 µM	[5]
Sodium dodecyl sulfate (SDS)/carbon paste electrode (CPE)	CV	10 to 80 µM	1.12 µM	[12]
	DPV	2 to 24 µM	0.39 µM	
Acetylene black/GCE	CV	0.01 to 2.5 μM	3.006 nM	[21]
Nickel hexacyanoferrate/Ni-Al layered double hydroxides (Ni-Al-LDH)/Au electrode	DPV	0.01 to 6.2 μM	0.0019 μM	[22]
NiCo-BTC metal–organic frameworks (MOFs)/nitrogen-doped graphene oxide nanoribbons (N-GO NRs)/GCE	Square-wave voltammetry (SWV)	0.01 to 800.0 μM	6.0 nM	[23]
MWCNTs/ZnO/SPCE	DPV	0.007 to 150.0 µM	0.002 µM	This work

**Table 2:** Determination of doxorubicin and dacarbazine in various samples using MWCNTs/ZnO/SPCE in 0.1 M phosphate buffer (pH 7.0) containing various additions of doxorubicin and dacarbazine (*n* = 5).

Sample	Spiked Concentration		Measured Concentration		Recovery		RSD (*n* = 5)	
Doxorubicin injection	Doxorubicin	Dacarbazine	Doxorubicin	Dacarbazine	Doxorubicin	Dacarbazine	Doxorubicin	Dacarbazine
	0	0	3.8 µM	-	-	-	3.2%	-
	2.0 µM	5.0 µM	5.7 µM	5.1 µM	98.3%	102.0%	2.4%	3.2%
	3.0 µM	7.0 µM	7.0 µM	6.8 µM	102.9%	97.1%	1.9%	2.7%
	4.0 µM	9.0 µM	7.9 µM	8.9 µM	101.3%	98.9%	2.8%	1.8%
	5.0 µM	11.0 µM	8.6 µM	11.4 µM	97.7%	103.6%	2.6%	2.5%
Dacarbazine injection	0	0	-	4.2 µM	-	-	-	2.7%
	5.0 µM	2.0 µM	4.9 µM	6.3 µM	98.0%	101.6%	3.0%	2.6%
	7.5 µM	3.0 µM	7.7 µM	7.1 µM	102.6%	98.6%	2.1%	2.0%
	10.0 µM	4.0 µM	9.9 µM	8.5 µM	99.0%	103.7%	2.8%	3.5%
	12.5 µM	5.0 µM	13.0 µM	9.0 µM	104.0%	97.8%	2.2%	1.9%

## Data Availability

The data presented in this study are available upon request to the corresponding authors.
